# Thymosin Beta 4 Protects Cardiomyocytes from Oxidative Stress by Targeting Anti-Oxidative Enzymes and Anti-Apoptotic Genes

**DOI:** 10.1371/journal.pone.0042586

**Published:** 2012-08-03

**Authors:** Chuanyu Wei, Sandeep Kumar, Il-Kwon Kim, Sudhiranjan Gupta

**Affiliations:** Division of Molecular Cardiology, Department of Medicine, College of Medicine, Texas A & M Health Science Center; Scott & White; Central Texas Veterans Health Care System, Temple, Texas, United States of America; UAE University, Faculty of Medicine & Health Sciences, United Arab Emirates

## Abstract

**Background:**

Thymosin beta-4 (Tβ4) is a ubiquitous protein with many properties relating to cell proliferation and differentiation that promotes wound healing and modulates inflammatory mediators. The mechanism by which Tβ4 modulates cardiac protection under oxidative stress is not known. The purpose of this study is to dissect the cardioprotective mechanism of Tβ4 on H_2_O_2_ induced cardiac damage.

**Methods:**

Rat neonatal cardiomyocytes with or without Tβ4 pretreatment were exposed to H_2_O_2_ and expression of antioxidant, apoptotic, and anti-inflammatory genes was evaluated by quantitative real-time PCR and western blotting. ROS levels were estimated by DCF-DA using fluorescent microscopy and fluorimetry. Selected antioxidant, anti-inflammatory and antiapoptotic genes were silenced by siRNA transfections in neonatal cardiomyocytes and effect of Tβ4 on H_2_O_2_-induced cardiac damage was evaluated.

**Results:**

Pre-treatment of Tβ4 resulted in reduction of the intracellular ROS levels induced by H_2_O_2_ in cardiomyocytes. Tβ4 pretreatment also resulted in an increase in the expression of antiapoptotic proteins and reduction of Bax/BCl_2_ ratio in the cardiomyocytes. Pretreatment with Tβ4 resulted in stimulating the expression of antioxidant enzymes copper/zinc SOD and catalase in cardiomyocytes at both transcription and translation levels. Tβ4 treatment resulted in the increased expression of anti-apoptotic and anti-inflammatory genes. Silencing of Cu/Zn SOD and catalase gene resulted in apoptotic cell death in the cardiomyocytes which was prevented by treatment with Tβ4.

**Conclusion:**

This is the first report that demonstrates the effect of Tβ4 on cardiomyocytes and its capability to selectively upregulate anti-oxidative enzymes, anti-inflammatory genes, and antiapoptotic enzymes in the neonatal cardiomyocytes thus preventing cell death thereby protecting the myocardium. Tβ4 treatment resulted in decreased oxidative stress and inflammation in the myocardium under oxidative stress.

## Introduction

Adverse cardiac remodeling is a detrimental process accountable for the development of various cardiac diseases including myocardial infarction, cardiac hypertrophy and heart failure. Although the mechanisms underlying the cardiac remodeling are multi-factorial, current evidences suggest that oxidative stress plays a critical role in the process. Oxidative stress is defined as an imbalance in antioxidant defense mechanism that elicits the production of reactive oxygen species (ROS) [1]–[4]. ROS are primarily characterized as oxygen based free chemical particles, if present in excess, causes contractile dysfunction and structural damage in the myocardium [5]. Therefore the balance between ROS production and removal of excess ROS are essential in maintaining the redox state and, homeostasis balance in the cell [6]. At the subcellular level, increased ROS levels can cause damage to nucleic acids and proteins leading to programmed cell death or apoptosis [7]–[9]. Thus, ROS mediated oxidative damage in cardiomyocytes is responsible for structural integrity of the myocardium.

It has been reported that increase in the levels of oxidative stress in the failing heart is primarily due to the functional uncoupling of the respiratory chain caused by inactivation of complex I in the mitochondria and considered to be a good source for ROS production [10], [11]. Another source would consider is the impaired antioxidant capacity that include superoxide dismutase (SOD), glutathione peroxidase (GSH-Px), and catalase (CAT) and considered as such as the first line of cellular defense against oxidative injury [12]. Accumulating evidences indicate that cardiac overexpression of Mn-SOD or CAT protects the heart from ischemic insult or myocardial infarction [13], [14].

Oxidative stress triggers pro-inflammatory signaling pathways that activate nuclear factor kappa B (NF-kB) and AP-1 transcription factors [15]. Previously, we and others have shown that NF-kB activation is associated with cardiac dysfunction, ventricular hypertrophy, and maladaptive cardiac growth [16]–[20]. The biochemical nexus between oxidative stress and inflammation represent an integral part in the pathophysiology of myocardial damage.

Thus, it is evident from our literature that oxidative damage remains a great challenge to promote significant myocardial damage and, numerous efforts have been made in the search of strategies to protect the heart against oxidative damage. In search of an ideal cardio-protective agent, Thymosin β4 (Tβ4) emerged as powerful candidate.

Tβ4, a G-actin sequestering molecule is primarily implicated in reorganizing actin cytoskeleton that needed for cell mobility [21]. Moreover, Tβ4 is present in all cells and body fluids and, has diverse biological function that includes tissue development, repair and pathology [21], [22]. Importantly, Tβ4 contributes a significant cardiac repair mechanism by activating integrin link kinase [23]–[25] and, has further shown to promote cardiac regeneration, epicardial cell migration and neovascularization [26], [27]. Our previous study demonstrated that treatment of Tβ4 restored the adverse cardiac remodeling (due to ischemic insult) by reducing inflammation, fibrosis and, activating ILK, PINCH and α-Parvin [27]. In the case of oxidative stress, Tβ4 has been shown to protect the cells by enhancing antioxidant enzymes and reducing caspase 9 activation in human corneal epithelial cells [28]–[30]. Under this setting, we recently have shown in cardiac fibroblast that Tβ4 has the target for SOD and catalase and thereby protect the cell from oxidative stress [31]. But the exact mechanism by which Tβ4 functions in the myocardium under oxidative stress and its effects on the cardiac myocytes is largely unknown.

The present study elucidates the protective mechanism of Tβ4 under oxidative stress using rat neonatal cardiac myocytes. We hypothesize that Tβ4 protect myocytes under oxidative stress by modulating antioxidant enzymes, apoptotic genes and pro-inflammatory genes. As for the limitation of our study, we used neonatal cardiomyocytes to study the protective effect of Tβ4 under oxidative stress conditions that may not mimic the changes in clinical conditions, and thus results using cultured cardiomyocytes should be interpreted carefully. An advantage of neonatal cardiomyocytes is the easy procedure for their isolation in contrast to adult cardiomyocytes, which are very sensitive to the concentration of Ca2+ in the medium. Moreover, the phenotype of cultured neonatal cardiomyocytes is very stable and their contractile profile very closely mimicking the adult cardiomyocytes. Experiments in isolated neonatal cardiomyocytes have generally reproduced the results on adult cardiomyocytes with a wide variety of interventions exploring the cellular and molecular mechanisms in oxidative stress.

## Materials and Methods

### Reagents

3-(4,5-Dimethylthiazol-2-yl)-2,5-diphenyltetrazolium bromide (MTT), hydrogen peroxide (H_2_O_2_) (Sigma), dimethyl sulfoxide (DMSO), dihydroethidium (DHE), dichlorofluorescein diacetate (DCF-DA), diaminofluorescein 2-diacetate (DAF-2DA), 3,3′-dihexyloxacarbocyanine iodide (DiOC_6_), and chloromethyl-X-rosamine (MitoTracker red) were purchased from Molecular Probes, Invitrogen, USA. Antibodies for Mn-SOD, Cu/Zn-SOD, Catalase, GAPDH, Bax, Bcl_2_, caspase-3 were purchased from Cell Signaling Technologies, USA Santa Cruz Biotechnologies (USA), Immuno-Rockland (USA). Protease inhibitor cocktail tablets were purchased from Roche GmbH, Germany. Dulbecco's Modified Eagle Medium (DMEM), non-essential amino acid cocktail, insulin, transferrin and selenium (ITS), and fetal Bovine Serum (FBS) were purchased from GIBCO, Invitrogen (USA). Thymosin β4 was supplied by RegeneRx Pharmaceutical.

### Cell culture and treatment

Primary cultures of cardiac myocytes were prepared from ventricles of 1–3-day-old Wistar rats as described previously [32]. In brief, cardiomyocytes were plated at a field density of 2.5×10^4^ cells per cm^2^ on coverslips, 6-well plates, 60-mm culture dishes, or 100-mm dishes as required with DMEM containing 10% FBS and supplemented with insulin, transferrin and selenium and bromo-deoxy-uridine. After 24 h, cells were serum deprived overnight before stimulation. A standardized dose of 100 µM H_2_O_2_ was used to induce oxidative stress in the *in vitro* system. To study the protective effects of Tβ4, cells were pretreated with Tβ4 2 hours prior to H_2_O_2_ challenge. The final concentration of Tβ4 used in this study was 1 µg/ml which was based on previous reports [28], [32].

### Detection of the cell viability

Cell viability of cardiac myocytes was measured quantitatively using MTT as described previously [31]. The absorbance was measured at 570 nm using a microplate reader (Molecular Devices, SpectraMax 250). The effect of Tβ4 was assessed on the H_2_O_2_ treated myocyte and the cytotoxicity curve was made and, expressed as percentage cell viability compared to control.

### Measurement of intracellular ROS levels

For measuring the levels of intracellular ROS, cardiac myocytes after treatments were incubated with 50 µM 2′,7′-dichlorodihydrofluorescein diacetate (H_2_DCFH-DA, Molecular Probes, Eugene, OR) at 37°C in the dark for 30 min as described previously [31].

### Confocal microscopy

For measuring the levels of intracellular ROS, cells were seeded on coverslips in 6-well plates and after treatments were incubated with 50 µM 2′,7′-dichlorodihydrofluorescein diacetate (H_2_DCFH-DA, Molecular Probes, Eugene, OR) at 37°C in the dark for 30 min as previously described [31]. Cells were then fixed and mounted on glass slides and observed under confocal laser scanning microscope (Fluoview FV1000) fitted with a 488 nm argon ion laser. Images were acquired using the F10-ASW 1.5 Fluoview software.

### Western blot analysis

Cardiac myocytes were treated with or without Tβ4 for 2 h before stimulated with 100 µM of H_2_O_2_. The cell lysate preparation, western blot analysis and image quantification were performed as described previously [31].

### RNA isolation and quantitative RT-PCR (q RT-PCR) analysis

Cardiomyocytes were treated with or without Tβ4 for 2 h followed by stimulation with H_2_O_2_ (100 µM) for up to 24 h. The preparation of RNA, 1^st^ strand cDNA synthesis and q RT-PCR was performed as described previously [31]. Analysis of relative gene expression was done by evaluating the real-time quantitative PCR data by 2^(−ΔΔCt)^ method as described previously by others [33], [34]. GAPDH or 18S was used as housekeeping gene.

### RNA interference and siRNA transfection

The gene silencing experiment using small interfering (si) RNA of Cu/Zn-SOD and Bcl_2_ was performed using predesigned double-stranded siRNA of the above from Sigma Life Science, Saint Louis MO, USA as described previously [31]. A scramble siRNA was used for negative control was also obtained from Sigma. In brief, cells were then transfected with 200 pmol of the siRNAs for Cu/Zn-SOD and Bcl_2_ or negative control siRNA using N-TER™ nanoparticle siRNA transfection system (Sigma) in accordance with the manufacturer's protocol. After 24 h of transfection, cells were treated and harvested to determine the transfection efficiency and effect of Tβ4 treatment on H_2_O_2_ treatment in the transfected cells.

### TUNEL staining

Quantification of TUNEL staining was done to study the extent of apoptotic cell death on transfected fibroblasts by *in situ* cell death detection kit (Roche Applied Science, Indianapolis, IN) as described previously [31].

### Statistical analysis

All experiments were performed at least three times for each determination. Data are expressed as means ± standard error (SE) and were analyzed using one-way analysis of variance and secondary analysis for significance with Tukey–Kramer post tests using Prism 5.0 GraphPad software (GraphPad, San Diego, CA, USA). A p value less than 0.05 was considered statistically significant.

## Results

### Tβ4 protects cardiomyocytes cells against H_2_O_2_-induced cell death

**Figure 1:**
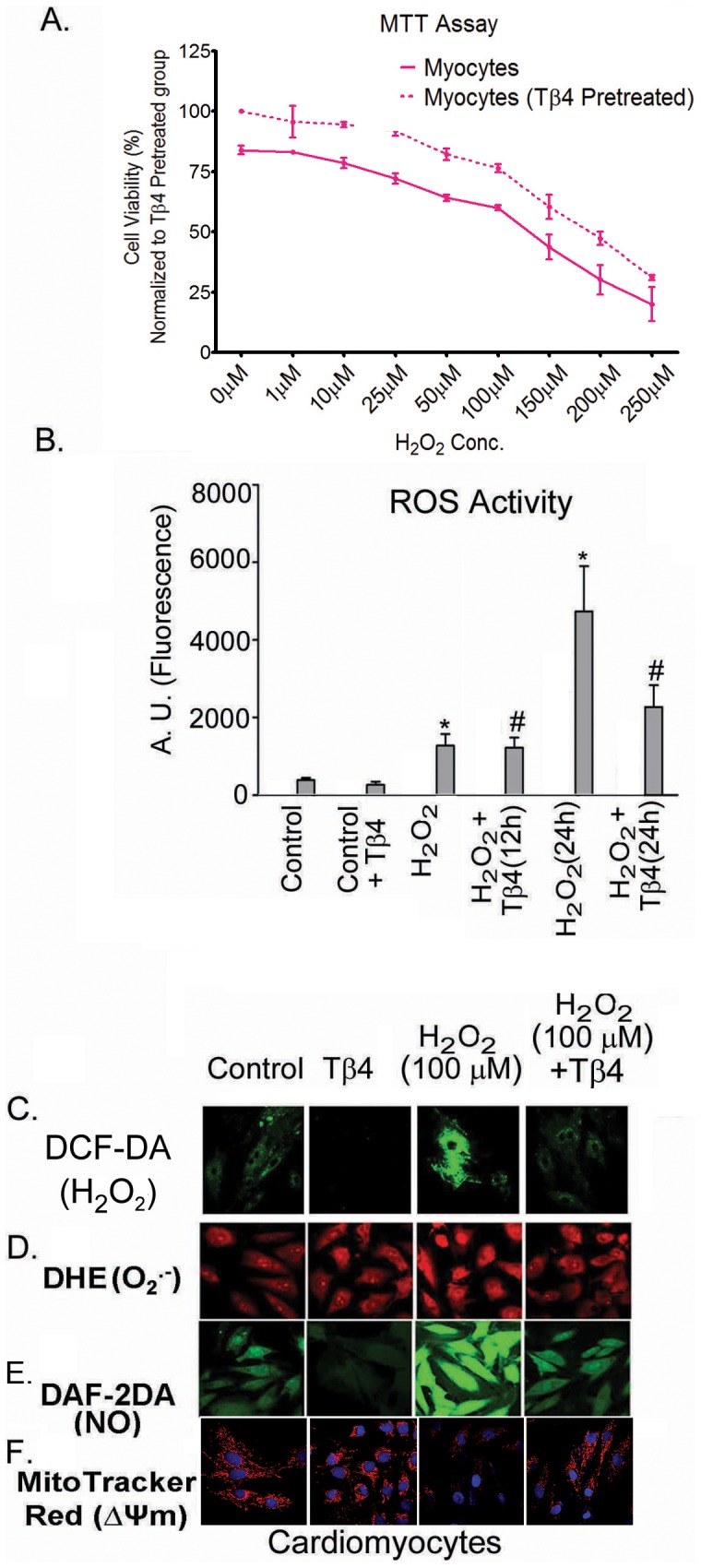
Effect of Tβ4 on cell viability in H_2_O_2_-treated cardiomyocytes. (**A**) The MTT assay was performed with increasing H_2_O_2_ concentration (1 to 250 µM) in presence (dotted lines) and absence (solid lines) of Tβ4 (1 µg/mL). Data represent means ± SEM of 3 individual experiments. (**B**). Effect of Tβ4 on generation of ROS in cardiomyocytes treated with H_2_O_2_ by fluorimetry. The graph represents the percentage of fluorescence positive cardiomyocytes upon staining with DCF-DA. Data represent the mean ± SE of at least three separate experiments. * means p<0.05 compared to the controls and # represents p<0.05 compared to the respective H_2_O_2_ treated group (**C**) Representative confocal laser scanning microscopy images of cardiomyocytes stained with DCF-DA showing the effect of Tβ4 on intracellular ROS upon treatment with H_2_O_2_. (**D**) Representative confocal laser scanning microscopy images of cells stained with DHE Red showing the effect of Tβ4 on generation of superoxide radicals upon treatment with H_2_O_2_ in cardiomyocytes. (**E**). Representative confocal laser scanning microscopy images of cells stained with DAF-2DA showing the effect of Tβ4 on generation of nitric oxide upon treatment with H_2_O_2_ in cardiomyocytes. (**F**). Representative confocal laser scanning microscopy images of cells stained with Mitotracker Red showing the effect of Tβ4 on loss of mitochondrial membrane potential upon treatment with H_2_O_2_ in cardiomyocytes.

The viability of cardiomyocytes was determined by MTT assay. Cardiomyocytes were treated with increasing doses of H_2_O_2_ and, cell viability was determined over a period of 24 hours. Our data showed that the 50% lethal dose (LD_50_) of H_2_O_2_ was between 150 and 250 µM (Figure 1A). Pretreatment with Tβ4 (1 µg/mL) prevented the myocyte cell death by 23.4% (p<0.05), compared to the H_2_O_2_-treated group indicating a protective role of Tβ4 in cardiomyocytes. The optimal sub-lethal concentration of H_2_O_2_ was determined and 100 µM H_2_O_2_ was used for the entire study.

### Tβ4 protects cardiomyocytes in H_2_O_2_-induced oxidative stress

Intracellular ROS levels in myocytes for 12 and 24 h post-H_2_O_2_ (100 µM) treatment were subsequently measured by fluorimetry and confocal microscopy analyses. There was an increase in ROS activity as indicated by increased fluorescence intensity of DCF-DA in the cardiomyocytes after H_2_O_2_ treatment (Figure 1B and 1 C). The fluorescence intensity of DCF-DA (indicative of oxidative burst) increased by 2.8-fold at 12 h (p<0.01) and 10.8-fold at 24 h (p<0.001), respectively, in the H_2_O_2_-treated group, compared to the untreated cardiomyocytes. Pretreatment with Tβ4 resulted in a 5.3% decrease at 12 h (n.s.) and 54.5% decrease at 24 h (p<0.01), respectively, compared to the H_2_O_2_-treated cells (Figure 1B and C) suggesting Tβ4 rescues cardiomyocytes from oxidative stress at later time-point. The quantifications of image intensities have been tabulated in Table 1.

**Table 1:** Image intensities (Arbitrary Units) showing the fluorescence intensities in cardiomyocytes upon staining with DCF-DA, DHE, DAF-2DA and MitoTracker Red.

S. No.	Staining	Control	Tβ4	H_2_O_2_	H_2_O_2_+Tβ4
1	DCF-DA	339±29	213±43*	792±53*	273±32^#^
2	DHE	592±71	602±68	904±50*	711±62^#^
3	DAF-2DA	414±39	184±37*	1311±92*	375±29^#^
4	MitoTracker Red	361±38	370±47	237±29*	349±24^#^

Data acquired from at least 15 fields taken from 3–4 different confocal images of the same treatment group and were quantified by using ImageJ Software.

* denotes p<0.05 compared to controls while # denotes p<0.05 compared to the H_2_O_2_-treated group.

### Tβ4 reduces the formation of superoxide radicals and nitric oxide in H_2_O_2_-induced oxidative stress in cardiomyocytes

H_2_O_2_ treatment induces a cascade of biochemical reaction in the cell leading to generation and accumulation of a variety of free radicals in the cells. We estimated the levels of superoxide and nitric oxide by using confocal microscopy. Our data revealed that there was an increase in the fluorescence intensity of DHE and DAF-2DA in H_2_O_2_ treated cells, an indicator of O_2_^.−^ and NO radicals, compared to unstimulated cells (Figure 1D and 1E). This increase in the fluorescence intensity of DHE and DAF-2A was significantly prevented by Tβ4 pretreatment. The quantifications of image intensities have been tabulated in Table 1.

### Tβ4 treatment protects mitochondrial membrane potential (ΔΨm) in oxide in H_2_O_2_-induced oxidative stress in cardiomyocytes

Oxidative stress is known to elicit depolarization of mitochondrial membrane potential. We evaluated the effect of Tβ4 on the mitochondrial membrane potential in H_2_O_2_ stimulated cardiomyocytes using MitoTracker Red by confocal microscopy. Our data revealed that there was loss of mitochondrial membrane potential as indicated by a decrease in the fluorescence intensity of MitoTracker Red H_2_O_2_ stimulated cell. Tβ4 treatment significantly restored the phenomenon (Figure 1F). The quantifications of image intensities have been tabulated in Table 1.

### Tβ4 upregulates antioxidant genes in cardiac myocytes under oxidative stress

**Figure 2:**
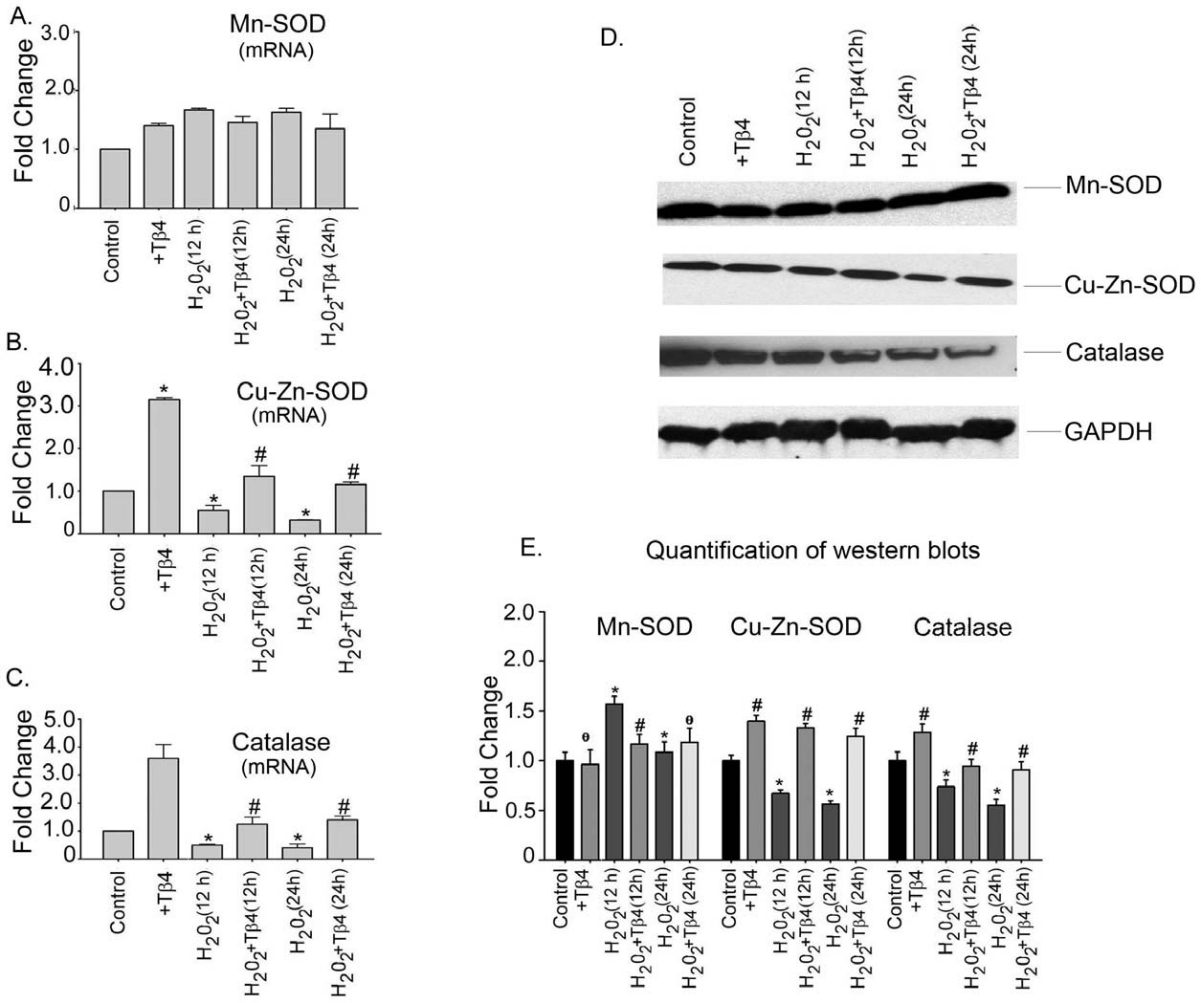
Effect of Tβ4 on anti-oxidative enzymes under oxidative stress in cardiomyocytes. (**A**) Relative fold change in the mRNA expression of Mn-SOD, (**B**) Cu/Zn-SOD and (**C**) Catalase, in cardiomyocytes treated with H_2_O_2_ in presence and absence of Tβ4. Data represent the means ± SE of at least three separate experiments. (**D**) Western blots showing the protein expression of Mn-SOD, Cu/Zn-SOD and catalase at 12 h and 24 h, respectively. GAPDH was used as internal loading control for the experiment. (**E**) Graph shows the relative fold change in the protein expression of Mn-SOD, Cu/Zn-SOD and catalase, respectively by densitometry. Data represent means ± SEM from 3 individual experiments. * denotes p<0.05 compared to controls while ^#^ denotes p<0.05 compared to the H_2_O_2_-treated group.

**Figure 3:**
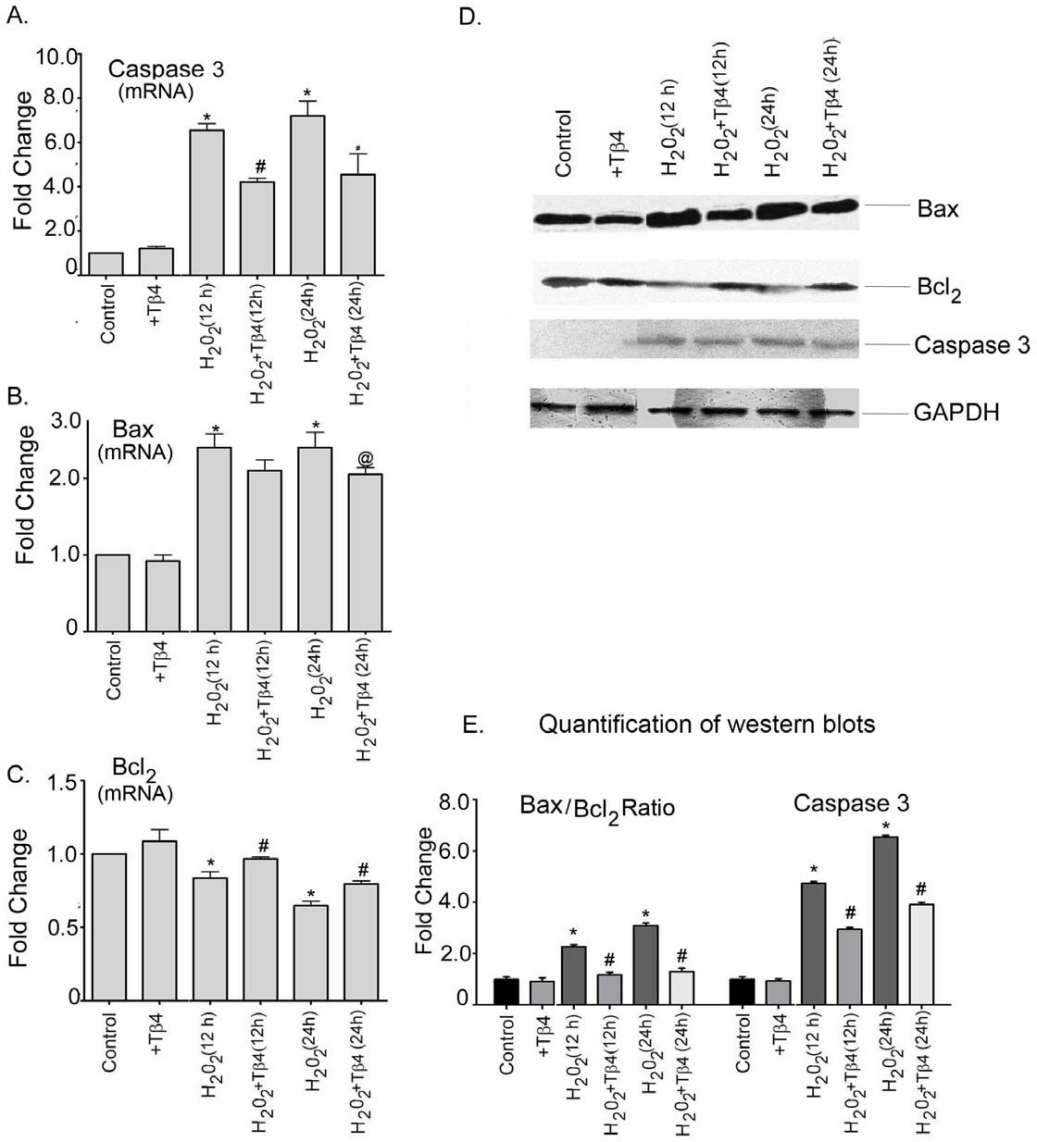
Effect of Tβ4 on pro-and anti-apoptotic proteins under oxidative stress in cardiomyocytes. Relative fold change mRNA expression of (**A**) Caspase-3, (**B**) Bax and (**C**) Bcl_2_. Data represent the means ± SE of at least three separate experiments. (**D**) Protein expression of Caspase-3, Bax and Bcl_2_ at 12 h and 24 h, respectively. GAPDH was used as loading control for the experiment. (**E**) Graph shows the relative fold change in the protein expression of Bax, Bcl_2_ and caspase-3, respectively by densitometry. Data represent means ± SEM from 3 individual experiments. * denotes p<0.05 compared to controls while ^#^ denotes p<0.05 compared to the H_2_O_2_-treated group and ^@^ means p = ns compare to the H_2_O_2_-treated group.

Since, oxidative stress alters the expression of antioxidant enzymes; we examined the mRNA expression of antioxidant genes, Mn-SOD, Cu/Zn-SOD and catalase in cardiomyocytes by q RT-PCR. In cardiomyocytes, the mRNA expression of Mn-SOD showed an initial increase and then a subsequent decline under H_2_O_2_ treatment. H_2_O_2_ treatment resulted in an increase in the Mn-SOD mRNA expression in 12 h by 1.8-fold and in 24 h by 1.7-fold, respectively (p<0.05), compared to the untreated cells. Tβ4 treatment did not significantly change the expression of Mn-SOD. Tβ4 pretreatment resulted in 1.07-fold (p = ns) and 1.04-fold (p = ns) decline in the mRNA expression of Mn-SOD at 12 h and 24 h, respectively, compared to H_2_O_2_-treated cells (Figure 2A). Neonatal cardiomyocytes treated with H_2_O_2_ showed a decline in the mRNA expression of Cu/Zn-SOD by 1.85-fold at 12 h (p<0.05) and 3.3-fold at 24 h (p<0.05), respectively, compared to the control. Compared to the H_2_O_2_-treated cells, pretreatment with Tβ4 upregulated the mRNA expression of Cu/Zn-SOD showing a 1.5-fold (p<0.001) and 2.9-fold (p<0.001) increase at 12 h and 24 h, respectively, suggesting that Tβ4 treatment reverts the Cu/Zn-SOD to normal (Figure 2B). The expression of antioxidant catalase which is a primary scavenger of H_2_O_2_ was also evaluated. The expression of catalase showed 2.12-fold and 2.77-fold (p<0.05) decline at 12 h and 24 h, respectively. Pretreatment with Tβ4 increased the mRNA expression of catalase by 1.4-fold (p<0.05) and 4.1-fold (p<0.05) at 12 h and 24 h, respectively, compared to H_2_O_2_-treated cells (Figure 3C) indicating that Tβ4 treatment prevented the depletion of antioxidant enzyme genes under oxidative stress (Figure 2C).

To evaluate the status of these antioxidant enzymes at protein levels, western blots were performed. Our data showed that the levels of Mn-SOD increased upon H_2_O_2_ challenge but did not change significantly upon treatment with Tβ4 The expression of Cu/Zn-SOD in H_2_O_2_-treated cells was reduced by 1.38-fold (p<0.05) and 1.5-fold (p<0.05) at 12 h and 24 h, respectively, compared to the untreated cells. The level of Cu/Zn-SOD was restored in Tβ4 treatment and showed a 2.0-fold (p<0.05) and 2.2-fold (p<0.05) increase at 12 h, and 24 h, respectively, compared to H_2_O_2_-treated cells. Similar changes were noted in the expression of antioxidant catalase, Tβ4 treatment per se increased the expression of catalase in the control cells by1.2-fold. There was 1.2-fold (p<0.05) and 1.85-fold (p<0.05) decrease in catalase treated with H_2_O_2_ at 12 h and 24 h, respectively, compared to the untreated cells. Pretreatment with Tβ4 resulted in an increase in the expression of catalase by 1.3-fold (p<0.05), and 1.8-fold (p<0.05) at 12 h and 24 h, respectively, compared to H_2_O_2_-treated cells. This indicates that Tβ4 preferentially upregulates the expression of Cu/Zn-SOD and catalase under oxidative stress (Figure 2D & E). The normalized quantification of Mn-SOD, Cu/Zn-SOD and catalase by western blotting is shown in the Figure 3E.

### Tβ4 protects cardiomyocytes from oxidative stress by increasing anti-apoptotic gene and reducing pro-apoptotic genes

Since oxidative stress leads to apoptotic cell death in cardiomyocytes, we evaluated the expression of pro- and anti-apoptotic genes. We determine the gene expression of Caspase-3, Bax and Bcl_2_ in H_2_O_2_ treated cells in the presence and absence of Tβ4. Under oxidative stress, there was a 6.6-fold (p<0.05), and 7.2-fold (p<0.05) increase in the mRNA expression of caspase3 in 12 h and 24 h, respectively compared to control. Tβ4 treatment resulted in 1.56-fold (p<0.05) and 1.58-fold (p<0.05) decrease in the mRNA expression of caspase3 at 12 h and 24 h treatment, respectively, compared to the H_2_O_2_-treated cardiomyocytes (Fig. 3A). Compared to the controls, H_2_O_2_ treatment resulted in an increase in the mRNA expression of Bax by 2.4-fold and 2.4 fold (p<0.05) at 12 h and 24 h, respectively (Figure 3 B). Tβ4 treatment reduced the increased Bax expression by 1.13-fold (p = n.s) and 1.2-fold (p<0.05) at 12 h and 24 h, respectively, compared to the H_2_O_2_-treated cells. Oxidative stress reduced the levels of anti-apoptotic gene Bcl_2_. Compared to the untreated groups, the mRNA expression of Bcl_2_ decreased by 1.56-fold (p<0.05) and 1.47-fold (p<0.05) at 12 h and 24 h, respectively, upon treatment with H_2_O_2_ (Figure 3 C). The reduced mRNA expression of Bcl_2_ under oxidative stress was reversed by pretreatment with Tβ4 by 1.14-fold (p<0.05) and 1.2-fold (p<0.05) at 12 h and 24 h, respectively, compared with the H_2_O_2_-treated cells (Figure 3 C).

At the translational level, H_2_O_2_ treatment resulted in a 4.7-fold (p<0.05), and 6.6-fold (p<0.05) increase in the expression of caspase3 at 12 h and 24 h treatment, respectively compared to the control. Tβ4 pretreatment in the H_2_O_2_ stimulated cells resulted in 1.6-fold (p<0.05) and 1.7-fold (p<0.05) decrease in the caspase3 protein expression at 12 h and 24 h treatment, respectively, compared to the H_2_O_2_-treated cells. (Figure 3 D). The normalized quantification of caspase-3 by western blotting is shown in the Figure 3 E. The Bax/Bcl_2_ ratio was also evaluated at protein level in cardiomyocytes. Our data showed that the Bax/Bcl_2_ ratio increased to 2.3-fold (p<0.05) and 3.0-fold (p<0.05) at 12 h and 24 h, respectively, under H_2_O_2_ treatment compared to the controls (Figure 3 D). Tβ4 treatment significantly reduced the increase in Bax/Bcl_2_ ratio by 1.9-fold (p<0.05) and 2.32-fold (p<0.05) at 12 h and 24 h, respectively, compared to the H_2_O_2_-treated group (Figure 3 E).

### Effect of Tβ4 treatment and analysis of NF-kB target genes by RT^2^ PCR array

To gain further insight into NF-kB-target genes, we performed q RT-PCR array. The data showed alteration of NF-kB family genes in H_2_O_2_ treated cardiomyocytes, compared to unstimulated cells. Furthermore, Tβ4 treatment restored those altered genes significantly. The list of NF-kB genes are shown in Table S1. Our data showed that H_2_O_2_ treatment induced upregulation of several NF-kB target genes, importantly, the following: TNFα, Irak1, Stat1, Tgfbr1, IkBα, IKKβ, Casp1, Rel, Egr1, NF-kB1, Tgfbr2, Rela, Ifnγ, Ccl2, Fasl, Il1β, IL-6 and Fadd. A list of selected NF-kB family genes is provided in Table S2.

### Validation of NF-kB target genes in cardiomyocytes

**Figure 4:**
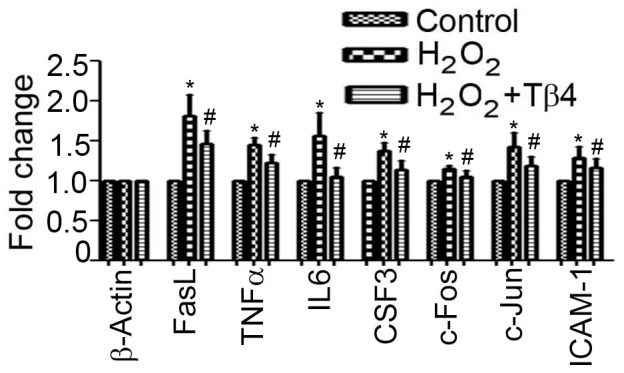
Effect of Tβ4 on pro-inflammatory molecules under oxidative stress in cardiomyocytes. Relative fold change in the mRNA expression of FasL, TNFα, c-Fos, c-Jun and ICAM1. Data represent the means ± SE of three separate experiments. * denotes p<0.05 compared to controls. # denotes p<0.05 compared to H_2_O_2_ group with Tβ4 treated group.

The expression of NF-kB target genes, FasL, TNFα, c-Fos, c-Jun and ICAM-1 were analyzed in H_2_O_2_ treated cardiomyocytes in the presence and absence of Tβ4. Our data showed that the expression of FasL, TNFα, c-Fos, c-Jun and ICAM-1 genes were increased by 1.45±0.07, 1.81±0.25, 1.14±0.03, 1.42±0.17and 1.29±0.12 fold (p<0.05), respectively, in H_2_O_2_ treated cardiomyocytes, compared to untreated cells. The Tβ4 treated showed significant restoration of the above genes by 1.22±0.1, 1.46±0.16, 1.05±0.06, 1.18±0.12 and 1.16±0.11fold (p<0.05), compared H_2_O_2_ treated cardiomyocytes (Figure 4).

### Tβ4 selectively upregulates Cu/Zn-SOD and Bcl_2_ genes in cardiac myocytes

**Figure 5:**
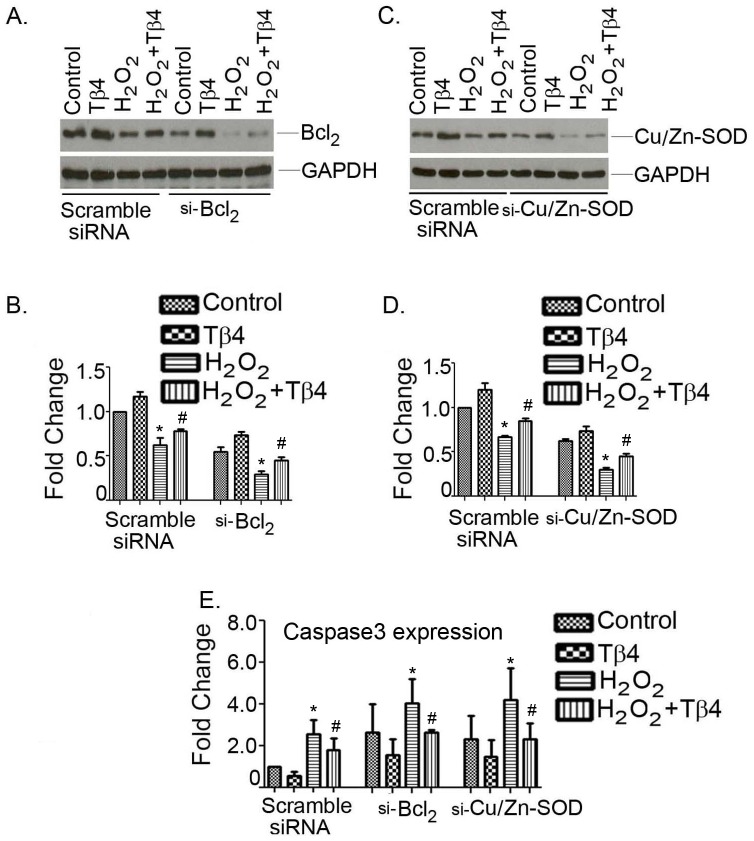
Effect of Tβ4 treatment after knocking down of Cu/Zn-SOD and Bcl_2_ in presence and absence H_2_O_2_-induced oxidative stress in cardiomyocytes. (**A**) Neonatal cardiomyocytes were transfected with scramble and Bcl_2_ siRNA, (**C**) scrambled and Cu/Zn-SOD siRNA in the presence and absence of Tβ4 under oxidative stress and Western blotting was performed using Bcl_2_ and Cu/Zn-SOD antibodies as probe. (**B and D**) Representative showing the quantification of panel A and C (**E**) Bar graph shows relative fold-change in the mRNA expression of caspase-3 in cardiomyocytes under similar experimental condition stated in A and B. Data represent the means ± SE of at least three separate experiments. * denotes p<0.05 compared to controls while ^#^ denotes p<0.05 compared to the H_2_O_2_-treated group with Tβ4 treated group.

We took knock-down approach to further validate the target molecule of Cu/Zn-SOD and Bcl_2_ by Tβ4. Both genes were knock- down in cardiomyocytes using their specific siRNAs and, were subsequently challenged with H_2_O_2_ in the presence and absence of Tβ4. The scramble siRNA were used as a control. Pretreatment with Tβ4 in scramble transfection enhanced the expression of Cu/Zn-SOD and Bcl_2_ under normal conditions (Fig. 5 A and 5 C). H_2_O_2_ treatment significantly downregulated the Cu/Zn-SOD and Bcl_2_ protein to 0.67±0.01 and 0.45±0.08-fold (p<0.05), respectively, compared to control. Tβ4 pretreatment for 24 h partly restored the expression of both Cu/Zn-SOD and Bcl_2_ to 0.84±0.03 and 0.74±0.01-fold (p<0.05), respectively, compared to H_2_O_2_ treated cells (Fig. 5 A and 5 C). Furthermore, the cardiomyocytes challenged with H_2_O_2_ in Bcl_2_ depletion showed further degradation of Bcl_2_ protein to 0.29±0.03-fold compared to H_2_O_2_ treated cells (p<0.05). Pretreatment partially recovered Bcl2 protein to 0.44±0.03-fold, compared t0 H_2_O_2_ treated cells. Likewise, the cardiomyocytes challenged with H_2_O_2_ in Cu/Zn-SOD depletion showed further degradation of Cu/Zn-SOD protein to 0.27±0.02-fold (p<0.05), compared to H_2_O_2_ treated cells. Pretreatment partially recovered Cu/Zn-SOD protein to 0.46±0.02-fold (p<0.05), compared t0 H_2_O_2_ treated cells. The quantification of western analysis was shown in Fig. 5 B and 5 D.

We then evaluated the expression of apoptotic marker gene, caspase-3 under the similar setting. The scramble transfection showed significant attenuation of caspase3 gene expression in Tβ4treated cells compared to H_2_O_2_ treated cells. The expression of caspase-3 increased to 2.56±0.69-fold (p<0.05) in H_2_O_2_ treated cell which was reduced by 1.80±0.54-fold upon pretreatment with Tβ4 (Fig. 5 E, left panel). Our data further showed that knocking down of both Cu/Zn-SOD and Bcl_2_ significantly enhance the caspases3 gene expression even in unstimulated cell. The expression of caspase3 was increased with the knockdown of Bcl_2_ by 2.65±1.3-fold in unstimulated cells. H_2_O_2_ treatment resulted in 4.04±1.16-fold increase and Tβ4 pretreatment showed 2.6±0.13-fold reduction of caspase3 expression in H_2_O_2_ treated cells (Fig. 5E, middle panel). The caspase3 gene expression was determined in Cu/Zn-SOD depleted cells. The expression of caspase3 was increased by 2.32±1.1 fold in unstimulated cells (Fig. 5E, right panel). The expression of caspase3 was further increased to 4.19±1.52 fold (p<0.05) with the knockdown of Cu/Zn-SOD gene by siRNA transfection. Tβ4 pretreatment showed 2.3±0.70-fold (p<0.05) reduction of caspase3 expression in H_2_O_2_ treated cells (Fig. 5E, right panel).

**Figure 6:**
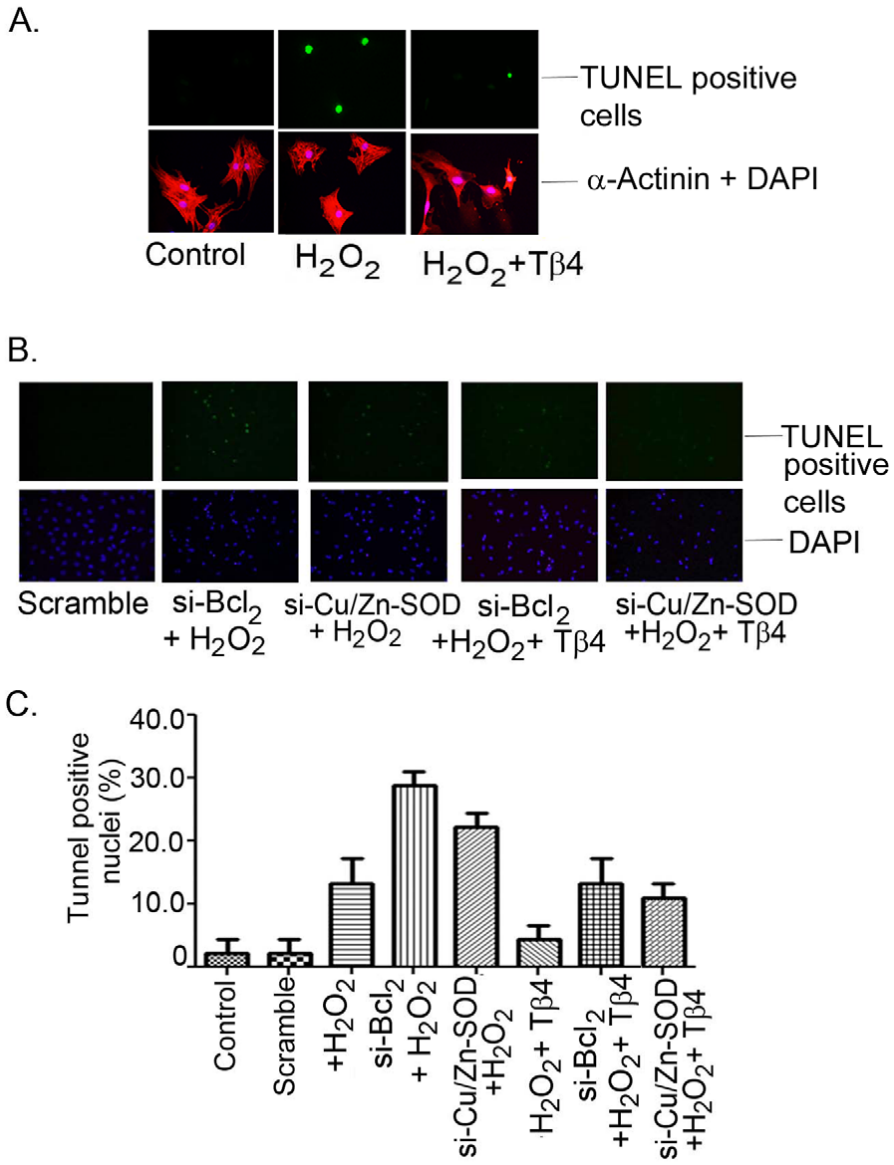
Representative fluorescent microscopy images of TUNEL staining in rat neonatal cardiomyocytes. (**A**) Bright TUNEL-positive images from control, H_2_O_2_ treated and H_2_O_2_ treated but pretreated with Tβ4 (upper panel). The lower panel showed the images of corresponding DAPI stained with α-actinin, a myocyte specific marker protein. (**B**) Representative fluorescent microscopy images showed the effect of Tβ4 treatment in the presence and absence of H_2_O_2_-induced oxidative stress on cardiomyocytes transfected with si-RNA-Bcl_2_ and siRNA-Cu/Zn-SOD vs. scrambled siRNA, respectively (**C**) Bar graph shows the percent TUNEL-positive nuclei under similar experimental condition. Data represent the means ± SE of at least three separate experiments. A total of 45 to 65 nuclei were counted for each observation. * denotes p<0.05 compared to controls while ^#^ denotes p<0.05 compared to the H_2_O_2_-treated group with Tβ4 treated group.

The TUNEL assay, performed under similar experimental conditions, showed increase in the TUNEL-positive nuclei under H_2_O_2_ treatment and, si-RNA knockdown of Cu/Zn-SOD, and Bcl_2_ further increased the TUNEL-positive cells. Representative fluorescence microscopy images showing TUNEL-positive nuclei (FITC-positive) of H_2_O_2_ treated in presence and absence of Tβ4 were shown in Figure 6 A and B. H_2_O_2_ treatment resulted in an increase of TUNEL-positive nuclei from 2.2±2.2% to 13.33±3.84% (p<0.05) in the scrambled si-RNA transfection. Both Bcl_2_ and Cu/Zn-SOD depleted cells challenge with H_2_O_2_ resulted further increase in the TUNEL-positive nuclei to 28.89±2.22% (p<0.05) and 22.22±2.622% (p<0.05), respectively (Figure 6 C). Pretreatment with Tβ4 in the H_2_O_2_ treated group resulted in a significant reduction in the TUNEL-positive nuclei to 4.4±2.2% (p<0.05) in scramble transfected cell, 13.3±3.84% (p<0.05) in si-RNA- Bcl_2_ and 11.11±2.22% (p<0.05) in si-RNA-Cu/Zn-SOD transfected cells, respectively (Figure 6 C). These results indicate that Tβ4 selectively targets Bcl_2_ and Cu/Zn-SOD genes to provide cardiac protection under oxidative stress.

## Discussion

The present study showed for the first time that Tβ4 protects cardiomyocytes under oxidative stress by upregulating antioxidant enzymes and reducing pro-apoptotic and pro-inflammatory genes. H_2_O_2_ elicits marked increment in intracellular ROS that promotes degradation of antioxidant enzymes (Cu/Zn-SOD and catalase) and activates pro-apoptotic (Bax and caspase3) and pro-inflammatory genes in cardiomyocytes. Increased ROS further advocate detrimental changes in cardiomyocytes leading to the loss of mitochondrial membrane potential and, subsequently increases the Bax/Bcl_2_ ratio favoring apoptosis. Pretreatment with Tβ4 showed significant attenuation of ROS activity and restoration of the above molecules and protecting cardiomyocytes from oxidative stress. Finally, we showed that knocking down of either Cu-Zn-SOD or Bcl_2_ in cardiomyocytes failed to protect the cells from oxidative stress in presence of Tβ4.

The myocardium has a complex mechanism to maintain the oxygen supply demand in response to diverse physiological and pathological stresses and, control the contractile function. The major pathological manifestation of oxidative stress is the generation of ROS that damage the cellular activity and function. It has become more apparent that the effect of oxidative stress in cardiac cells predisposes the condition that lead to adverse cardiac remodeling including cell death, myocardial hypertrophy and contractile dysfunction [6], [35], [36]. Cardiomyocytes are the major “bulk” in the myocardium and primarily governs the contractile function. Any sort of stress will have a serious impact on cardiomyocytes and affect various signaling cascades that ultimately lead to dysfunction. In an attempt to protect these cells under oxidative stress, we tested the efficacy of Tβ4 in cardiomyocytes which is currently undetermined. Our results indicate that cardiomyocytes pretreated for 2 h with Tβ4 increases the cell viability under oxidative stress suggest that Tβ4 contributes a crucial role in the cardio-protection under oxidative stress.

Oxidative stress and ROS have been implicated in triggering cell death. Following a one-, two- or three-electron reduction, O_2_ may generate successively O_2_ **^.^** ^−^ (superoxide radical), H_2_O_2_ or OH^−^ (hydroxyl radical). ROS are able to oxidize biological macromolecules such as DNA, protein and lipids [37], [38]. Superoxide dismutase (SOD) converts O_2_ **^.^** ^−^ into H_2_O_2_ and the latter can generate OH^−^ in the presence of Fe^2+^ cations (Fenton reaction). It should be noted that nitric oxide (NO) can also be oxidized into reactive nitric oxide species, which may show behavior similar to that of ROS. In particular the combination of NO and O_2_ **^.^** ^−^ can yield a strong biological oxidant, peroxynitrite that is more detrimental to the cells [10], [39]. In our study, we showed that treatment of Tβ4 restored all H_2_O_2_ induced free radical generation in cardiomyocytes suggesting a protective role in this setting. One of the traditional hallmarks of ROS-initiated cell death is mitochondrial dysfunction and energy depletion [40], [41]. Several mechanisms can impair energy production in cardiac mitochondria, including damage to the electron transport chain and phosphorylation apparatus, mtDNA injury, opening of the mitochondrial permeability transition pore (MPTP), the loss of the mitochondrial membrane potential (ΔΨm) and, the concomitant drop in ATP production [42], [43]. Dysfunction of mitochondrial machinery in the heart releases apoptotic signaling molecules e.g. cytochrome c and may cause an irreversible injury to the mitochondria [44]. Our data showed significant decrease in ΔΨm which was prevented by pretreatment with Tβ4.

Tβ4 is very effective in reducing intracellular ROS in H_2_O_2_-treated cardiomyocytes. Our study is the first to show that the attenuation of ROS is mediated by restoring Cu/Zn-SOD and catalase, the two important antioxidant enzymes. Another relevant antioxidant that loses function upon oxidation is Mn-SOD. Although, both Mn-SOD and Cu/Zn-SOD have been reported to play a crucial role in protecting the cardiac cells from oxidative damage by scavenging ROS [13], [45] but, we found that Tβ4 upregulated the expression levels of Cu/Zn-SOD in cardiomyocytes. Catalase, which was directly responsible for H_2_O_2_ clearance, was upregulated by Tβ4 both at mRNA and protein level in the presence of H_2_O_2_ stimulus indicating that Tβ4 preferentially targets catalase in the cardiomyocytes which enable effecting scavenging of the H_2_O_2_ from the system. Also it was worth notice that even though the protein and gene expression levels of both catalase and Cu/Zn-SOD were increased by Tβ4, this peptide upregulated the gene encoding the former more efficiently in cardiomyocytes. Furthermore, oxidative stress promotes apoptotic cell death by lowering Bax/Bcl_2_ ratio. In our study, we showed that Tβ4 reduced the intracellular ROS levels in cardiomyocytes and prevents cell death by restoring Bax/Bcl_2_ ratio and inhibiting the activation of caspase3. This observation supports our previous observation using cardiac fibroblast [31] but, contrast to the previous reported by Sosne G *et al* where they did not observe any change in Bax/Bcl_2_ expression [30]. We did not know the reason for this but, the use of different cell type may accountable for this altered phenomenon.

To confirm the target of Cu/Zn-SOD and Bcl_2_ by Tβ4 in order to protect the cardiomyocytes from oxidative stress, we selectively knocked down these molecules and determined the efficacy of Tβ4 under oxidative stress. We found that Tβ4 prevented cell death by specifically targeting Cu/Zn-SOD and Bcl_2_ molecules in H_2_O_2_ treated cardiomyocytes. But, when these molecules were knocked down in the cell, Tβ4 failed to protect the cells from apoptosis. These data led us to convey the message that Tβ4 may provide cardiac protection under oxidative stress by restoring Cu-Zn SOD and Bcl_2_ levels in the myocardium.

Our study also indicates that Tβ4 protects the cardiomyocytes from oxidative stress by attenuating pro-inflammatory genes regulated by NF-kB. It is evident that ROS activation often triggers NF-kB translocation and thereby promotes pro-inflammatory response [46], [47] As mentioned previously, ROS are toxic in cells and damage the cellular integrity, it is therefore, critical to make a balance of ROS production in order to prevent further oxidative damage. In this setting, our study further indicates that Tβ4 protects the cardiomyocytes from oxidative stress by attenuating the pro-inflammatory genes regulated by NF-kB. Taken together, our data validate and re-established a potential role Tβ4 as an anti-inflammatory molecule which may provide a new therapeutic module for cardiac protections under oxidative stress. Future studies may aim to delineate the interaction or association between NF-kB and Tβ4 in the context of NF-kB transcriptional regulatory circuit and anti-inflammatory properties in the cardiac cells.

In conclusion, we demonstrated that Tβ4 protects the myocardium from oxidative stress by reducing ROS activity via re-establishing the antioxidant enzyme levels, Cu/Zn-SOD and catalase and, further attenuating Bax and caspase3 levels and restoring Bcl_2_ as well. Our results not only offered more mechanistic explanation about the protective mechanism of Tβ4 but also supported the need to further investigate the use of this small molecule in protecting the myocardium against oxidative damage in variety of disease condition where ROS has been implicated to play a damaging role like cardiac hypertrophy and heart failure.

### Therapeutic implication

Our findings are relevant in the clinical settings as many studies have shown that depletion of anti-oxidants in the heart makes it more vulnerable to damage especially under ischemia and under high pro-oxidant condition. Although, we did not investigate adult rat cardiomyocytes, but, many studies have shown that primary cultured neonatal rat cardiomyocytes were useful models to investigate cardio-protective effects. Future studies are, therefore, warranted to examine the effect of Tβ4 under the similar setting. We believe that Tβ4 is a better therapeutic target as it has the ability to enhance the expression of the selected antioxidant and anti-inflammatory genes, thereby, alleviating the damage to the myocytes under oxidative stress. These possibilities regarding the mechanisms whereby Tβ4 modulates the above molecules need to be further tested experimentally in future studies.

## Supporting Information

Table S1 NF-kB RT^2^ PCR array Cells were treated with H_2_O_2_ in the presence and absence of Tβ4 and NF-kB RT^2^ PPCR array was performed using a kit from SA Bioscience according to the manufacturer's protocol.
(DOCX)

Table S2 Selected NF-kB family genes.
(DOCX)
