# Supplementary material for: Thymosin Beta 4 Protects Cardiomyocytes from Oxidative Stress by Targeting Anti-Oxidative Enzymes and Anti-Apoptotic Genes
Source: PLoS One. 2012 Aug 3;7(8):e42586. doi: 10.1371/journal.pone.0042586 (PMC3411836; doi:10.1371/journal.pone.0042586)
Supplement: Table S1 — NF-kB RT2 PCR array Cells were treated with H2O2 in the presence and absence of Tβ4 and NF-kB RT2 PPCR array was performed using a kit from SA Bioscience according to the manufacturer's protocol. (DOCX) [file pone.0042586.s001.docx]

| **Symbol** | **Gene Name** | **Tβ4-treated group vs. Control**  **(Fold Regulation)** | **H_2_O_2_-treated group vs. Control**  **(Fold Regulation)** | **H_2_O_2_ + Tβ4-treated group vs. H_2_O_2_-treated group**  **(Fold Regulation)** |
| --- | --- | --- | --- | --- |
| Akt1 | V-akt murine thymoma viral oncogene homolog 1 | -7.1 | 5.3 | -2.4 |
| Atf1 | Activating transcription factor 1 | -2418.7 | 7.1 | -2.4 |
| Atf2 | Activating transcription factor 2 | -1176.3 | 7.8 | -2.9 |
| Bcl10 | B-cell CLL/lymphoma 10 | 7.3 | -75.1 | 1.9 |
| Bcl3 | B-cell CLL/lymphoma 3 | 4.2 | -238.9 | -49.2 |
| C3 | Complement component 3 | -38431.5 | 3.9 | -1606.8 |
| Card10 | Caspase recruitment domain family, member 10 | -38.6 | 14.6 | 1.6 |
| Casp1 | Caspase 1 | -8.2 | 6.5 | -71.5 |
| Casp8 | Caspase 8 | -1.6 | 7.7 | -4.7 |
| **Ccl2** | Chemokine (C-C motif) ligand 2 | -3082.7 | 2.8 | -8.2 |
| Cflar | CASP8 and FADD-like apoptosis regulator | -661.7 | 7.4 | -3.3 |
| Chuk | Conserved helix-loop-helix ubiquitous kinase | -14.8 | 7.4 | -3.0 |
| Crebbp | CREB binding protein | -5.0 | 8.2 | -2.8 |
| Csf2 | Colony stimulating factor 2 (granulocyte-macrophage) | -10.9 | 12.7 | 1.9 |
| Csf3 | Colony stimulating factor 3 (granulocyte) | -1.5 | 42.2 | 4.4 |
| Lpar1 | Lysophosphatidic acid receptor 1 | -897.6 | 5.5 | -5.0 |
| Egr1 | Early growth response 1 | -37.0 | 10.0 | 3.9 |
| F2r | Coagulation factor II (thrombin) receptor | -1686.7 | 4.8 | -1.5 |
| Fadd | Fas (TNFRSF6)-associated via death domain | -21.6 | 8.8 | -2.3 |
| **Fos** | FBJ osteosarcoma oncogene | -2.6 | 8.5 | 1.5 |
| Gja1 | Gap junction protein, alpha 1 | -1016.9 | 8.2 | 1.9 |
| Htr2b | 5-hydroxytryptamine (serotonin) receptor 2B | 25.6 | 14.9 | -11.3 |
| **Icam1** | Intercellular adhesion molecule 1 | -1296.1 | 6.1 | -1.9 |
| Ifna1 | Interferon-alpha 1 | 1562.9 | 4.1 | 1.1 |
| Ifng | Interferon gamma | 79.3 | 94.4 | 1.1 |
| Ikbkb | Inhibitor of kappa light polypeptide gene enhancer in B-cells, kinase beta | -903.9 | 9.6 | -3.1 |
| Ikbke | Inhibitor of kappa light polypeptide gene enhancer in B-cells, kinase epsilon | -166.6 | 10.7 | -3.1 |
| Ikbkg | Inhibitor of kappaB kinase gamma | -313.0 | 9.4 | -3.1 |
| Il10 | Interleukin 10 | -9.4 | 13.2 | -2.3 |
| Il1a | Interleukin 1 alpha | -149.1 | 6.1 | -6.3 |
| Il1b | Interleukin 1 beta | -2836.7 | 4.0 | -117.8 |
| Il1r1 | Interleukin 1 receptor, type I | -4576.4 | 5.8 | -3.0 |
| **Il6** | Interleukin 6 | -224.4 | 182.3 | -1.0 |
| Irak2 | Interleukin-1 receptor-associated kinase 2 | -5.1 | 10.7 | -3.7 |
| Irf1 | Interferon regulatory factor 1 | -21.4 | 9.1 | -2.6 |
| **Jun** | Jun oncogene | -17.0 | 7.8 | -1.3 |
| Kcnh8 | Potassium voltage-gated channel, subfamily H (eag-related), member 8 | 11.2 | 19.6 | 1.1 |
| Tbk1 | TANK-binding kinase 1 | 1.3 | 8.6 | -5.0 |
| Tnfsf14 | Tumor necrosis factor (ligand) superfamily, member 14 | 5.5 | 14.9 | -52.3 |
| Tlr7 | Toll-like receptor 7 | -12.0 | 6.7 | -288.0 |
| Irak1 | Interleukin-1 receptor-associated kinase 1 | -2210.3 | 7.7 | -3.0 |
| Lta | Lymphotoxin alpha (TNF superfamily, member 1) | -2.5 | 44.3 | -1.9 |
| Ltbr | Lymphotoxin beta receptor (TNFR superfamily, member 3) | -44.0 | 7.3 | -2.4 |
| Map2k3 | Mitogen activated protein kinase kinase 3 | -19.8 | 8.9 | 1.6 |
| Map3k1 | Mitogen activated protein kinase kinase kinase 1 | -749.6 | 9.3 | -7.6 |
| Mapk3 | Mitogen activated protein kinase 3 | -5367.4 | 6.2 | -3.9 |
| Myd88 | Myeloid differentiation primary response gene 88 | -1992.0 | 7.6 | -4.7 |
| Nalp12 | NACHT, leucine rich repeat and PYD containing 12 | -1.3 | 4.7 | -1.0 |
| Nfkb1 | Nuclear factor of kappa light polypeptide gene enhancer in B-cells 1 | -2352.5 | 136.2 | -2.4 |
| Nfkbia | Nuclear factor of kappa light polypeptide gene enhancer in B-cells inhibitor, alpha | -2998.4 | 49.5 | -3.5 |
| Pcaf | P300/CBP-associated factor | -2521.4 | 6.8 | -5.2 |
| Ppm1a | Protein phosphatase 1A, magnesium dependent, alpha isoform | -3104.2 | 6.8 | -1.8 |
| Eif2ak2 | Eukaryotic translation initiation factor 2-alpha kinase 2 | -4269.9 | 5.0 | -2.3 |
| Raf1 | V-raf-leukemia viral oncogene 1 | -3848.3 | 7.7 | -2.3 |
| Rel | V-rel reticuloendotheliosis viral oncogene homolog (avian) | -357.1 | 89.3 | -5.4 |
| Rela | V-rel reticuloendotheliosis viral oncogene homolog A (avian) | -2759.1 | 69.1 | -2.4 |
| Nfkb2 | Nuclear factor of kappa light polypeptide gene enhancer in B-cells 2, p49/p100 | -427.6 | 8.6 | -3.6 |
| Ippk | Inositol 1,3,4,5,6-pentakisphosphate 2-kinase | -243.9 | 9.3 | -1.8 |
| Ripk2 | Receptor-interacting serine-threonine kinase 2 | -2225.6 | 6.4 | 1.4 |
| Slc20a1 | Solute carrier family 20 (phosphate transporter), member 1 | -982.3 | 6.5 | -1.4 |
| Smad3 | SMAD family member 3 | -385.3 | 9.1 | -1.0 |
| Smad4 | SMAD family member 4 | -3590.6 | 6.6 | -1.9 |
| Stat1 | Signal transducer and activator of transcription 1 | -2683.7 | 5.6 | -3.2 |
| **Tgfbr1** | Transforming growth factor, beta receptor 1 | -2759.1 | 7.0 | -7.5 |
| **Tgfbr2** | Transforming growth factor, beta receptor II | -6562.4 | 6.8 | -3.8 |
| Tlr1 | Toll-like receptor 1 | -596.3 | 9.6 | -130.7 |
| Tlr2 | Toll-like receptor 2 | -2402.0 | 7.1 | -13.4 |
| Tlr3 | Toll-like receptor 3 | -652.6 | 9.2 | -6.5 |
| Tlr4 | Toll-like receptor 4 | -776.0 | 7.3 | -8.9 |
| Tlr6 | Toll-like receptor 6 | -306.6 | 10.8 | -9.1 |
| Tlr9 | Toll-like receptor 9 | -837.5 | 8.9 | -648.1 |
| **Tnf** | Tumor necrosis factor (TNF superfamily, member 2) | -1428.2 | 4.6 | -82.1 |
| Tnfrsf10b | Tumor necrosis factor receptor superfamily, member 10b | -152.2 | 12.5 | 2.2 |
| Tnfrsf1a | Tumor necrosis factor receptor superfamily, member 1a | -5634.2 | 6.8 | -2.5 |
| Tnfrsf1b | Tumor necrosis factor receptor superfamily, member 1b | -530.1 | 8.7 | -6.9 |
| Cd40 | CD40 molecule, TNF receptor superfamily member 5 | -433.5 | 9.3 | 1.4 |
| Tnfsf10 | Tumor necrosis factor (ligand) superfamily, member 10 | -1.6 | 46.5 | -1.2 |
| **Faslg** | Fas ligand (TNF superfamily, member 6) | -1.2 | 68.6 | 3.1 |
| Tnip2 | TNFAIP3 interacting protein 2 | -1120.6 | 8.0 | -3.5 |
| Tollip | Toll interacting protein | -814.6 | 8.8 | -1.9 |
| Tradd | TNFRSF1A-associated via death domain | -1428.2 | 8.1 | -2.9 |
| Traf2 | Tnf receptor-associated factor 2 | -929.3 | 8.7 | -1.9 |
| Traf3 | Tnf receptor-associated factor 3 | -843.4 | 8.3 | -1.4 |
| Zap70 | Zeta-chain (TCR) associated protein kinase | -1.2 | 53.1 | 1.1 |

Selected Genes from the array for further validation

| **Symbol** | **Fold Regulation**  **Only Tβ4** | **Fold Regulation**  **Only H_2_O_2_** | **Fold Regulation**  **H_2_O_2_ + Tβ4** |
| --- | --- | --- | --- |
| Il6 | -224.4111 | 182.2784 | -1.007 |
| Nfkb1 | -2352.5342 | 136.2394 | -2.362 |
| Ifng | 79.3413 | 94.3532 | 1.1019 |
| Rel | -357.0544 | 89.2636 | -5.4264 |
| Rela | -2759.1344 | 69.0706 | -2.4284 |
| Faslg | -1.1728 | 68.5935 | 3.0738 |
| Zap70 | -1.1728 | 53.0765 | 1.1019 |
| Nfkbia | -2998.4475 | 49.5221 | -3.4581 |
| Tnfsf10 | -1.5911 | 46.5271 | -1.2311 |
| Lta | -2.4967 | 44.3235 | -1.9319 |
| Csf3 | -1.5263 | 42.2243 | 4.4076 |
| Kcnh8 | 11.2356 | 19.5622 | 1.1019 |
| Tnfsf14 | 5.5022 | 14.9285 | -52.3457 |
| Htr2b | 25.6342 | 14.9285 | -11.3137 |
| Card10 | -38.5859 | 14.6213 | 1.5583 |
| Il10 | -9.3827 | 13.1775 | -2.2974 |
| Csf2 | -10.8528 | 12.7286 | 1.9185 |
| Tnfrsf10b | -152.2185 | 12.4666 | 2.1886 |
| Tlr6 | -306.5545 | 10.7779 | -9.1261 |
| Irak2 | -5.0982 | 10.7034 | -3.6553 |
| Ikbke | -166.5718 | 10.7034 | -3.0738 |
| Egr1 | -37.014 | 9.9866 | 3.8906 |
| Tlr1 | -596.3436 | 9.6465 | -130.6896 |
