# Supplementary material for: Thymosin Beta 4 Protects Cardiomyocytes from Oxidative Stress by Targeting Anti-Oxidative Enzymes and Anti-Apoptotic Genes
Source: PLoS One. 2012 Aug 3;7(8):e42586. doi: 10.1371/journal.pone.0042586 (PMC3411836; doi:10.1371/journal.pone.0042586)
Supplement: Table S2 — Selected NF-kB family genes. (DOCX) [file pone.0042586.s002.docx]

| **Symbol** | **Fold Regulation**  **Only Tβ4** | **Fold Regulation**  **Only H_2_O_2_** | **Fold Regulation**  **H_2_O_2_ + Tβ4** |
| --- | --- | --- | --- |
| Il6 | -224.4111 | 182.2784 | -1.007 |
| Nfkb1 | -2352.5342 | 136.2394 | -2.362 |
| Ifng | 79.3413 | 94.3532 | 1.1019 |
| Rel | -357.0544 | 89.2636 | -5.4264 |
| Rela | -2759.1344 | 69.0706 | -2.4284 |
| Faslg | -1.1728 | 68.5935 | 3.0738 |
| Zap70 | -1.1728 | 53.0765 | 1.1019 |
| Nfkbia | -2998.4475 | 49.5221 | -3.4581 |
| Tnfsf10 | -1.5911 | 46.5271 | -1.2311 |
| Lta | -2.4967 | 44.3235 | -1.9319 |
| Csf3 | -1.5263 | 42.2243 | 4.4076 |
| Kcnh8 | 11.2356 | 19.5622 | 1.1019 |
| Tnfsf14 | 5.5022 | 14.9285 | -52.3457 |
| Htr2b | 25.6342 | 14.9285 | -11.3137 |
| Card10 | -38.5859 | 14.6213 | 1.5583 |
| Il10 | -9.3827 | 13.1775 | -2.2974 |
| Csf2 | -10.8528 | 12.7286 | 1.9185 |
| Tnfrsf10b | -152.2185 | 12.4666 | 2.1886 |
| Tlr6 | -306.5545 | 10.7779 | -9.1261 |
| Irak2 | -5.0982 | 10.7034 | -3.6553 |
| Ikbke | -166.5718 | 10.7034 | -3.0738 |
| Egr1 | -37.014 | 9.9866 | 3.8906 |
| Tlr1 | -596.3436 | 9.6465 | -130.6896 |
